# Administrative Data Linkage in Brazil: Potentials for Health Technology Assessment

**DOI:** 10.3389/fphar.2019.00984

**Published:** 2019-09-23

**Authors:** M Sanni Ali, Maria Yury Ichihara, Luciane Cruz Lopes, George C.G. Barbosa, Robespierre Pita, Roberto Perez Carreiro, Djanilson Barbosa dos Santos, Dandara Ramos, Nivea Bispo, Fabiana Raynal, Vania Canuto, Bethania de Araujo Almeida, Rosemeire L. Fiaccone, Marcos E. Barreto, Liam Smeeth, Mauricio L. Barreto

**Affiliations:** ^1^Faculty of Epidemiology and Population Health, Department of Non-communicable Disease Epidemiology, London School of Hygiene and Tropical Medicine, London, United Kingdom; ^2^Nuffield Department of Orthopaedics, Rheumatology and Musculoskeletal Sciences (NDORMS), Center for Statistics in Medicine (CSM), University of Oxford, Oxford, United Kingdom; ^3^Centre for Data and Knowledge Integration for Health (CIDACS), Instituto Gonçalo Muniz, Fundação Osvaldo Cruz, Salvador, Brazil; ^4^Institute of Public Health, Federal University of Bahia (UFBA), Salvador, Brazil; ^5^University of Sorocaba–UNISO, São Paulo, Brazil; ^6^Centro de Ciências da Saúde, Federal University of Recôncavo of Bahia (UFRB), Salvador, Brazil; ^7^Department of Management and Incorporation of Health Technology, Ministry of Health (DGITS/MS), Brasília, Brazil; ^8^Department of Statistics, Federal University of Bahia (UFBA), Salvador, Brazil; ^9^Department of Computing, Federal University of Bahia (UFBA), Salvador, Brazil; ^10^Institute of Health Informatics, University College London, London, United Kingdom

**Keywords:** administrative data, Brazil, data linkage, epidemiological studies, health technology assessment, record linkage

## Abstract

Health technology assessment (HTA) is the systematic evaluation of the properties and impacts of health technologies and interventions. In this article, we presented a discussion of HTA and its evolution in Brazil, as well as a description of secondary data sources available in Brazil with potential applications to generate evidence for HTA and policy decisions. Furthermore, we highlighted record linkage, ongoing record linkage initiatives in Brazil, and the main linkage tools developed and/or used in Brazilian data. Finally, we discussed the challenges and opportunities of using secondary data for research in the Brazilian context. In conclusion, we emphasized the availability of high quality data and an open, modern attitude toward the use of data for research and policy. This is supported by a rigorous but enabling legal framework that will allow the conduct of large-scale observational studies to evaluate clinical, economical, and social impacts of health technologies and social policies.

## Introduction

Health technology assessment (HTA), also known as healthcare technology assessment or medical technology assessment, is the systematic evaluation of the properties, intended and unintended effects and/or impacts of health technologies and interventions (Batarrita, 1999; Banta, 2009). It is an investigative process that evaluates the clinical (effectiveness and safety), economical (cost or cost-effectiveness), ethical, and social consequences of using new or existing technologies in health with the main goal of improving “value for money” in health care (Banta, 2009; Kristensen et al., 2009). Health technologies can be any intervention in health with the aim of promoting health and prevent, diagnose, or treat disease, examples include: drugs, devices, procedures, and the organizational, educational, informational, and support systems within which health care is delivered to the population (Banta, 2009). HTAs are useful to a wide range of decision makers in healthcare: government policy makers, insurance companies, and other payers, industries, planners, administrators, clinicians, and patients. Although the goal of HTA is to support policy decision and not sole knowledge generation, it must be carried out with integrity and using solid scientific methods to yield valid results (Banta, 2009).

In the mid-1980s, a new constitution in Brazil ruled that health is a right of all citizens and a duty of the State. This was the starting point for the building up of the Brazilian unified national health system [Sistema Único de Saúde (SUS), literally the “Single Health System”]. Since that moment, the Brazilian government showed strong interest in HTA, particularly after organizing and leading, in collaboration with Pan American Health Organization/World Health Organization (PAHO/WHO), on a HTA conference in Brasilia, Brazil (Banta, 2009; Guimarães, 2014). The discussions involved the political aspects of HTAs, including the questionable effectiveness of technologies used in health, cost and cost-effectiveness, and the process of technology transfer. In the years from 2000 to 2008, substantial actions were taken in HTA: (1) Several seminars and consultations were held. (2) Key institutional changes were made at the Ministry of Health (MoH), including the formation of the Department of Science and Technology (DST); the Secretariat of Science, Technology and Strategic Inputs (SSTSI); and the Commission for the Incorporation of Technologies (the Commission). Later, the DST joined the International Network of Agencies in HTA. (3) Policies were developed by the federal government to encourage HTA and its application for clinical, management, and policy decisions. The SSTSI was then given the mandate for policy implementation in relation to pharmaceuticals within the SUS. At the same time, a process flow for incorporating technologies under the auspices of SUS and Supplementary Health System (SHS) was established, which was later redefined. Furthermore, the SSTSI was assigned to oversight the Commission; to evaluate and recommend the incorporation, alteration, or exclusion of products for the SUS and SHS procedure lists; to propose the revision of therapeutic guidelines; and to order and carry out HTA-specific studies. (4) Several academic institutions, such as the Federal University of Rio de Janeiro and the State University of Sao Paulo, developed HTA research initiative and the Brazilian Network for HTA (REBRATS), coordinated by MoH, was established by the adhesion of HTA groups scattered throughout universities, medical schools, and teaching hospitals (Banta, 2009; Guimarães, 2014).

In December 2011, the National Committee for Health Technology Incorporation into SUS (CONITEC) was created by the Brazilian Government through a Federal Law (12.401/2011) and regulated by Presidential Decree (7.646 from December 21, 2011). Aiming to provide greater agility, transparency, and efficiency to the health technologies, incorporation or disinvestment processes, CONITEC has set a new milestone to health management with innovative precepts in HTA in Brazil. With the objective of providing administrative, technical, and scientific support to CONITEC, the Executive Secretariat of CONITEC was created, which is in charge of the Department of Management and Incorporation of Health Technologies (DGITS) of the SSTSI of the MoH. The actions developed by DGITS, assisted by a network of national institutions (hospitals and universities), which are partners of CONITEC, have been successful in promoting public consultations, making decisions about claims, and acquiring health technology products and services (Guimarães, 2014). Currently, most of the HTA procedures within the REBRATS realm make predominant use of secondary sources of information, especially studies in the field of meta-analyses and other publications, for the incorporation of technologies (Guimarães, 2014).

Brazil has a long tradition of keeping records of health-related information for administrative purposes owing to the establishment of SUS, its informatics department (DATASUS), and the substantial progress made toward Universal Health Coverage (UHC) (Paim et al., 2011). Despite the efforts made by the MoH to harmonize the recording of information, a great disparity exists among various health institutions related to data collection processes. In addition, individual data collected by different health services (for example, hospital and mortality registries) lack unique key identifiers for individuals, hence, combining these data sources is not a trivial task. These factors, in addition to the technological infrastructure and skilled human resource constraints, have limited the use of routinely collected data to generate evidence to support clinical and policy decisions and to answer important epidemiological questions.

However, in the past decade, several big data and record linkage initiatives, different record linkage software packages (for example, Reclink, AtyImo, and CIDACS-RL) (Camargo and Coeli, 2000; Pita et al., 2018), and international collaborations on research and capacity building have emerged. The use of record linkage technology to integrate data that are not available in a single data set by supplementing information from other data sources and/or validate information collected in one data source has made the conduct of health outcomes research possible in the Brazilian setting (Camargo and Coeli, 2000; Pinto et al., 2017). The HTA field in Brazil will benefit from such institutional and technological advances in data processing and analysis to produce evidence on the (cost-)effectiveness and safety of health technologies, as well as their impact of social, economic, and health policies. Hence, the objective of this manuscript was to review the main health care and socioeconomic databases, recent advances in the use of big data and data linkage tools; and to highlight the potentials and challenges of using secondary data and data linkage for health outcomes and policy research, as well as HTA.

This manuscript is organized as follows: the section *Databases Used in Health Outcomes/Policy Research in Brazil* describes the major databases used in health outcomes/policy research in Brazil, the section *Data Linkage* introduces important concepts in data linkage, the section *Data Linkage Initiatives in Brazil* describes major initiatives in creation of data centers and development of data linkage in Brazil, the section *Record Linkage Tools Developed and/or Used in Brazil* summarizes record linkage algorithms developed/used in Brazilian databases, the section *Challenges and Opportunities* highlights major challenges in the use of secondary data for health research in Brazil, and the section *Conclusion* concludes the manuscript.

## Databases Used in Health Outcomes/Policy Research in Brazil

In Brazil, the main databases storing health-related information are generated from SUS (de Mello Jorge et al., 2010; Paim et al., 2011; Souza et al., 2016). These databases can be classified into: (1) epidemiological (such as the Live Births Information System/SINASC; the Mortality Information System/SIM; the Information System for Notifiable Diseases/SINAN), which are used for surveillance, evaluation, and research to address public health questions; (2) administrative (such as the Outpatient Information System/SIA-SUS and the Hospital Information System/SIH-SUS), which are used for accounting and control of the production of the services provided; and (3) clinical, which are used to store clinical data on patients for future reference (Souza et al., 2016).

In addition, other government sectors also generate and manage data on demographic and socio-economic characteristics of the Brazilian population. For example, the Ministry of Social Development maintains an electronic database (“Cadastro Único,” CadUnico) for provision of social services, such as the Conditional Cash Transfer Program (BFP) and the Housing Program (MCMV). These databases, in combination with others, have been used to study social determinants of health and evaluations of social policies on health (Rasella et al., 2013; Nunes et al., 2016; Machado et al., 2018). **Table 1** summarizes some details about the main databases publicly available from SUS and other governmental sources in Brazil.

**Table 1:** Databases From the Brazilian Public Health System (SUS) and Other Government Sources.

Abbreviation	Year	Registers
CadUnico	2003	Individuals and their socio-economic characteristic applying for social benefits.
BFP	2003	Individuals receiving BF payments.
SINASC	1990	All births in Brazil including the type of pregnancy and delivery.
SIM	1975	All deaths in Brazil including ICD-10 cause of death.
SINAN	1993	Diseases of compulsory notification using ICD-10 codes.
SIH-SUS	1993	Patient admissions in the network of public hospitals under SUS.
SIA-SUS	1995	Outpatient visits by SUS.
APAC-SIA	1996	High-cost ambulatory procedures and high-cost medicines.
RHC	1967	Cancer patients in (public or private) hospitals responsible for oncology care.
RCBP	1967	Cancer patients in centers located mostly in major cities.
SISMAMA	2004	Information about breast and gynaecological cancer screening.
SI-PNI	1973	Dispensed immunobiologicals.
SIAB-SUS	1998	Home visits, and medical and nursing care performed in households and health unit
SISLAB-GAL	2008	Laboratory test including cases of Compulsory Notification.
NOTIVISA	2008	Spontaneous reports of suspected cases of Adverse Drug Events.
SNGPC	2007	Dispensing movement data (inputs and outputs) of the drugs subject to special control and antimicrobials.
SINITOX	1980	Cases of intoxication and poisoning.
PFPB	2004	Medication dispensation in the FPB Program.

### Cadastro Único

Large social inequalities and poverty are major historical characteristics of Latin America and Brazil, in particular (Bértola and Williamson, 2017). To reduce poverty and inequalities, Brazil has implemented several social protection policies including the Conditional Cash Transfer Program—”Bolsa Familia Program,” the housing program “Minha Casa Minha Vida,” and the access to water program “Cisterns,” among others. In 2003, the Brazilian government created the unified registration for social programs, Cadastro Único (CadUnico), to facilitate implementation and to support decisions related to applications for any of the available social protection programs (Mostafa and Silva, 2007). CadUnico is an electronic database comprising individual records of 114 million people (57% of the Brazilian population, until 2015). It has information on the household characteristics and individual members, who applied to any of the 20 social benefits (from 2004 onward) and those who received any social benefit (since 2001), including detailed demographic, economic, and social conditions of the household (Mostafa and Silva, 2007; Rodrigues, 2017). It is continuous for new applicants, and those already registered have to update the information every 2 years. For those who had their benefits turned down but want to reapply and those who are already receiving a benefit, the information update is mandatory. The extensive coverage of this social registry, the availability of individually identified data, and the possibility of linking them to other health care databases, such as SIM/SINASC/SINAN, allow for designing individual level longitudinal studies to evaluate the impact of social protection programs on health outcomes (such as diseases, hospitalizations, and deaths) (Paim et al., 2011; Rasella et al., 2013; Nunes et al., 2016; Machado et al., 2016a) and has inspired the development of the 100 Million Brazilian Cohort (Pinto et al., 2017; Pita et al., 2018).

### Bolsa Família Program

The Brazilian government introduced the largest conditional cash transfer program in the developing world called “Bolsa Família Program” in 2003 as a merger of the pre-reform cash transfers (Lindert et al., 2007). The aim was to reduce current poverty and inequality, by providing a minimum level of income for extremely poor families and to break the inter-generational transmission of poverty by conditioning these transfers on beneficiary compliance with human capital requirements. The conditionalities include: 1) children aged 7 to 17 years have to attend a minimum of 85% schooling days; 2) children up to 7 years of age must complete vaccination and growth monitoring; and 3) beneficiary families with pregnant women, nursing mothers, or children younger than 7 years should follow a health and nutrition agenda (pre- and post-natal care, vaccination, and health and nutrition surveillance). It is implied that making the benefits conditional on “positive” behaviors can further increase the chances of breaking out of the poverty cycle through increased education or improved health. The program also seeks to help empower BFP beneficiaries by linking them to other complementary services, such as health and education (Lindert et al., 2007; Paes-Sousa et al., 2011).

BFP targets were identified through geographic and household assessment methods based on per capita household income. Geographic targeting is applied at federal and municipal levels where as family eligibility is determined based on household registry data that was collected locally and transmitted to the central database, the CadUnico (Lindert et al., 2007). The cash transfers are intended for poor and extremely poor households, with additional payments when a household include children up to 17 years of age (up to two payments per family), or pregnant women (up to nine monthly payments) or lactating women (up to six monthly payments). The original income ceilings for eligibility to the BFP program were set at a fixed monthly per capita household income of R $100 (US $48) for poor families and R $50 (US $25) for extremely poor families. To account for increases in the cost of living, the thresholds were increased in 2006 to R$120 (US $57) for poor families and R$60 (US $29) for extremely poor families (Machado et al., 2018). Additional adjustments were made in 2009 (R$140 for poor and R$70 for extremely poor families) and in 2014 (R$154 for poor and R$77 for extremely poor families). BFP covers 23% of the Brazilian population with the benefits ranging from $18 to a maximum of $175 per month. The mother, when present, must receive the monthly payment on behalf of the whole family (Paiva et al., 2013).

The BFP has attracted significant attention both in Brazil and beyond. As such, several studies have been conducted to evaluate the impact of this program on several health-related outcomes, such as poverty reduction (Soares et al., 2006), inequalities (Soares et al., 2006), crime (Chioda et al., 2016; Machado et al., 2018), leprosy incidence (Nery et al., 2014), and child mortality and hospital admissions (Rasella et al., 2013). Information recorded for each household include date of start of the benefit, period of receipt, and amount of monthly cash transferred (Ministro da Cidadania, 2017). This database, in combination with CadUnico, provides socio-economic information for nearly half of the Brazilian population in the lower income category.

### SINASC

The Live Births Information System (Sistema de Informação Sobre Nascidos Vivos [SINASC]), created in 1990 by the MoH, contains vital information on live births in Brazil with the most significant characteristics about the newborn, the mother, the pregnancy, and the delivery. The system includes consolidated data since 1994 and operates with a standardized model of the birth certificate (the Declaration of Live Birth, DNV, a “declaração de nascido vivo”), a legal document completed by the health provider who assisted the delivery and then collected by health secretariat (Frias et al., 2014).

SINASC includes information on place of delivery (hospital or home), the mother who gave birth (including name, age, place of residence, marital status, education, number of children, and number of previous live and still births), the pregnancy (number of prenatal appointments, length of gestation, type of pregnancy: singleton or twin, type of delivery); and the newborn (gestational age, birth weight, sex, ethnicity, the presence and type of birth anomalies for live and stillbirths, and 1- and 5-min APGAR score for live births) (da Saúde, 2011). SINASC uses the International Classification of Disease Version 10 (ICD-10) for coding congenital defects (do Nascimento et al., 2018).

Data must be uploaded by SUS’s local level manager, the Municipal Health Secretariat, who are also responsible for processing, consolidating, evaluating, and analyzing these data to support decision-making at local level. Data completeness and coverage are very high, with more than 90% completeness for most variables at country level and capturing 97% of Brazilian registered births (Pedraza, 2012; Oliveira et al., 2015). However, this coverage is heterogeneous within the country, with large variations among the states and some with low percentages particularly those located in the North and Northeast regions. In addition, under-registration of births is still common in some regions of the country and inconsistency of records in variables, such as mother’s education, race, and number of prior childbirths, is still high in North and Northeast regions (Oliveira et al., 2015; Hunter and Sugiyama, 2018). SINASC, in combination with SIM (the Mortality Information System) and SIH (the Hospital Information System), has been used to study the impacts, burden, and/patterns of diseases (Paixao et al., 2018), pregnancy-related hospitalizations (Moura et al., 2018), impact of socio-economic inequalities on prenatal consultation (Mallmann et al., 2018), factors affecting neonatal mortality (Kropiwiec et al., 2017; Paixao et al., 2018), the use of ICD-10 coding system on congenital disease ascertainment (do Nascimento et al., 2018). SINASC and SIM/SIH also provide data that are used as parameters in the HTA studies.

### SIM

The Mortality Information System (Sistema de Informação sobre Mortalidade [SIM]) was the first subsystem of health information created in 1975 and managed by MoH, containing records of all deaths in Brazil, including fetal deaths. These records are based on the standard death certificate (called the Declaration of Death; DO, “declaração de óbito”), a required legal document, and fetal death certificate collected by the state health secretariat, which contributes to the improvement in the registration of data (Oliveira et al., 2015). Information recorded include: name, date of birth, date of death, sex, ethnicity, educational level, marital status, occupation, place of death, type of health service where death occurred (hospital, another type of health unit, home, or elsewhere), ICD-10 code causes of death (main and secondary) and comorbidities (up to two) (Victora and Barros, 2001). The correct coding of the cause of death, according to ICD-10, is of great importance for the good quality of SIM data. Like SINASC, the coverage in SIM is heterogeneous within the country, with large variations among the states and some with low percentages particularly those located in the North and Northeast regions (Victora and Barros, 2001).

SIM, with SIH/SINASC, has been used in linkage studies (Kropiwiec et al., 2017; Paixao et al., 2018), characterization of trends and regional patterns in (cause-specific) maternal and infant mortality (Victora and Barros, 2001), trends and disparities in cancer mortality (Alves et al., 2009; Girianelli et al., 2014; Braga et al., 2017; Prado da Fonseca et al., 2018), among others. SIM has good coverage and quality, and death characterization. However, delays in data processing, under-reporting of deaths, high numbers of ill-defined cause of death, variation of the quality and coverage in different geographical areas, as well as incorrect filling of death certificates, are some of the limitations (Victora and Barros, 2001; Santos et al., 2008).

### SINAN

The Notifiable Diseases Information System [Sistema de Informação de Agravos de Notificação (SINAN)] was implemented gradually and disorderly from 1993. In 1998, it became mandatory to feed the system with data on diseases of compulsory notification, such as tuberculosis, leprosy, human immunodeficeincy virus/acquired immune deficiency syndrome (HIV-AIDS), leishmaniasis, dengue, and Zika. There is a national list of these diseases; however, some states could also include their own specific health problems or outbreaks. Information on disease, using ICD-10 code, is collected through forms filled by health professionals who attend patients with suspected diseases. There are three documents: 1) Individual Notification Form (FIN), filled at the hospital when there is a suspicion of obligatory notifiable disease, outbreak, or new/unknown diseases. This form is followed by: 2) Negative Notification, when there is no disease confirmation; 3) Individual Investigation Form (FII) on identification of the source of infection and transmission mechanism. Each disease record includes different variables, but all include: name, sex, date of birth, place of residence, years of education, date of onset, and clinical aspects of the disease such as symptoms, laboratory tests, disease severity, and sometimes the outcome of the treatment (Paixao et al., 2018). SINAN facilitates the study determinants of obligatory notifiable diseases; indicates the risks of diseases; and facilitates standardization of procedures, investigations, and forms for notifiable diseases. However, there is under-reporting especially of patients from private practices, delay in data processing and correction, and long and complicated information flow (Laguardia et al., 2004).

SINAN, linked to SIM and/or SINASC, has been used to evaluate maternal and child health outcomes (Paixao et al., 2018), incidence and prevalence studies (Tanaka et al., 2017), tuberculosis (Oliveira et al., 2012a; Saraceni et al., 2018), and HIV studies after linkage with other administrative databases SISCEL (Laboratory Tests Control Systems) and SICLOM (Medication Logistics Control System) administrative databases made available in 2000 and 2006, respectively (Saraceni et al., 2018). SICLOM database covers all people living with HIV and receiving ART (antiretroviral therapy), both in public and private health care sectors. SISCEL database, on the other hand, covers only those people living with HIV who had CD4 and viral load tests conducted in public laboratories (Saraceni et al., 2018).

### SIH

The Hospital Information System [Sistema de Informações Hospitalares (SIH)] is the national administrative database established in 1991 and comprises information on patient admissions in the network of public hospitals under SUS and private hospitals contracted by the SUS. It has information on over 75% of the country’s hospitalizations that are covered/funded by the SUS. Hospitalizations in SUS require completion of a standard form (authorization for hospitalization) that captures patients’ personal data, symptoms, and ICD-10 codes of the initial diagnosis. This form and other information recorded by the SIH-SUS on diagnoses, treatment, test results, and billing are standardized throughout Brazil. The resulting data are checked and validated by local health authorities and subsequently transmitted to regional and national levels (Coelho et al., 2016).

Variables recorded include: sex, age, number of hospitalizations, the total amount and value of reimbursed hospital services, days and average length of stay, mortality, among others (Melione and Jorge, 2008; Coelho et al., 2016). SIH has high agility, good morbidity information; it is regularly submitted to audit and payment review; and also allows for monitoring of surveillance epidemiology. However, it mainly covers the public health system which accounts for about 70% of the total admissions and it is constantly changing (Medeiros et al., 2005; Melione and Jorge, 2008; Coelho et al., 2016; Machado et al., 2016a). Although it was created with an administrative purpose, it has been frequently used to monitor population health states and observational studies of adverse drug events (Martins et al., 2018) and health care costs (Quarti Machado Rosa et al., 2018).

### SIA-SUS

The Outpatient/Ambulatory Information System of SUS [Sistema de Informações Ambulatoriais do Sistema Único de Saúde (SIA-SUS)] was implemented throughout the country in 1995 and records outpatient visits through the Ambulatory Production bulletin (BPA). Data processing occurs in a decentralized way in which each state and municipality, duly qualified, can register, program, process, and pay for the production of its health facilities under its management. For the generation of information, SIA uses some basic systems, such as the SUS Procedure Chart Management System (SIGTAP), capture application, such as magnetic ambulatory production bulletin, and authorization of magnetic ambulatory procedures. Both capture applications allow recording of basic-, medium-, and high-complexity care procedures. SIA is widely used for HTA studies, since it provides, in addition to the quantitative procedure performed in the SUS, the cost of these procedures for SUS (Machado et al., 2016b).

### APAC-SIA

The System of High Complexity Procedures Authorization (APAC-SIA) is a SIA sub-system, established in 1996, constituted by individual registers of high-cost ambulatory procedures and high-cost medicines for specific diseases such as biologics (Brito et al., 2005; Peres et al., 2016; Machado et al., 2016b). Access to high-cost medicines is *via* SUS’s Specialized Component of Pharmaceutical Service through a form that comprises useful clinical information of the patient (Machado et al., 2016b). This database contains information on name, national health card number, age, sex, mother’s name, address, main procedure code and name, amount of procedures, brief description of diagnosis, ICD-10 code, concomitant diseases, and health care professional number and register code.

The High Complexity Oncology Procedures Authorizations (APAC-ONCO) database contains additional information, including diagnosis date, primary cancer site; histopathology description and final diagnosis; ICD-10 topography; lymph node invasion (yes/no); metastasis locations; tumor, node, metastasis (TNM) stage; and stage by different system. It also records information on previous treatment and current treatment (surgery, chemotherapy, or radiotherapy), including description and start date, scheme, planned duration, and irradiated areas. APAC-ONCO has been used in cancer studies after linkage with other databases, such as SIM (Machado et al., 2016b; Peres et al., 2016), SIH, the breast cancer screening information system (SISMAMA) (Peres et al., 2016), and the hospital-based cancer registry (RCBP) (Peres et al., 2016).

### Cancer Information Systems

The cancer registry is a service for collecting, storing, analyzing, interpreting, and systematically disseminating cancer data and includes: 1) The Hospital Cancer Registry (RHC) for recording information about cancer patients seen in a particular hospital (public or private) responsible for oncology care. RHC has administrative purposes, such as estimation of future demand, equipment needs, and human resources. It is considered highly representative of the baseline population and is useful to determine diagnosis efficiency, stage at diagnosis, and treatment. 2) The Population-Based Cancer Registry (RCBP) which was established in 1967 with 26 centers located mostly in major cities. It monitors the frequency of new cancer cases between regions and over time by collecting diagnoses from different sources (clinicians and pathologists) or the death data (when the main cause is cancer). Both registries record socio-demographic information about the patient (age, education level, marital status and place of residence), family history of cancer, source and year of referral, date of diagnosis, diagnosis and previous cancer treatment, characteristics of the tumor (synchronous tumor and laterality), date of first appointment and initiation of the treatment, type of treatment received, stage at diagnosis and tumor evolution after the treatment, and cost of diagnosis and treatment (Ferreira et al., 2017).

The National Cancer Institute (INCA) branch of the MoH in partnership with DATASUS has also implemented the Information System for the Control of Breast Cancer (SISMAMA), an online tool that register information about breast and gynaecological cancer screening. In Brazil, mammograms are encouraged by SUS targeting the female population older than 50 years and has been performed every 2 years, or annually in the case of altered clinical examinations (Cecilio et al., 2015). Women presenting with familial history are encouraged to undergo annual screening of the breasts (Lima-Costa and Matos, 2007). It is estimated that 50% of Brazilian women older than 50 years have had at least one mammography in their life (Lima-Costa and Matos, 2007; Anderson et al., 2011). SISMAMA was conceived as a management tool that capture, organize, and make available data about the population tested; test results (mammograms and ultrasounds, and breast cytopathology and histopathology); follow-up of abnormal cases; the quality of the services; as well as other essential information generated in the course of providing screening tests (Passman et al., 2011). Data collection begins in the primary care setting, typically with a physician’s order for a screening or diagnostic mammogram. Mammography results are classified using the Breast Imaging Reporting and Data System (BI-RADS) developed by the American College of Radiology (ACR). SISMAMA has been used in several studies after linkage with SIM, SIA-APAC, and SIH-SUS (Freire et al., 2015; Peres et al., 2016; Tomazelli et al., 2018a; Tomazelli et al., 2018b).

### SI-PNI

The National Immunization Program Information System [Sistema de Informação do Programa Nacional de Imunização (SI-PNI)] contains records on dispensed immunobiologicals. It was developed by PNI in partnership with DATASUS and is comprise several subsystems: 1) the Information System of the Immunization Program Assessment (SI-API) which provides data on vaccination coverage (routine and campaigns), dropout rate, immunization control bulletins. API can be used by the federal, state, regional, and municipal levels; 2) the Immunobiological Inventory and Distribution Information System (SI-EDI) which controls the supply and distribution of immunobiologicals at the state and federal levels; 3) the Information System of adverse events following vaccination (SI-EAPV), which allows the vigilance of adverse events after administration of the vaccine; 4) the Information System of the Instrument Evaluation Program (SI-PAIS) and the Information System of the Evaluation Program of the Supervision Instrument in Vaccine Room (SI-PAISSV), which contribute for standard evaluation profile and fast delivery of tabulated results; 5) the Information System for the Assessment of Immunobiologicals Used (SI-AIU), which evaluates the lost and utilized doses; 6) the Information System of the Reference Center for Special Immunobiological (SI-CRIE) which informs adverse events and utilization of special immunobiologicals (da Nóbrega et al., 2010).

SI-PNI enables quantitative analysis of vaccination coverage by vaccine type, doses given, and dropout rate throughout the country by age group, time, and geographical area (Assis Moura et al., 2018). Within SI-PNI, it is also possible to perform monthly follow-up of vaccination activities regarding the quantity of distributed and applied doses, coverage, and adverse events post-vaccination (EAPV). SI-PNI uses single identifying number shown on the “National Health Card” [Cart ao Nacional de Sa ude (CNS)], hence, the vaccinated and their origins can be identified, allowing to find unvaccinated ones and give them a dose. Linkage to different national databases enables conduct of observational studies on vaccine effectiveness (Domingues and Teixeira, 2013; Sato, 2015). In addition, SI-PNI can be used as parameter to assess and modeling economics evaluation of new vaccines.

### SIAB

The Basic Health Care Information System [Sistema de Informação da Atenção Básica (SIAB)] was created in 1998 by DATASUS, in conjunction with the co-ordination of community health/health care Secretariat (COSAC/SAS). It assists monitoring and evaluation of activities carried out by the community health agents (ACS), aggregating and processing the data from the home visits, as well as the medical and nursing care performed in households and health unit (Da Silva and Laprega, 2005; Frias et al., 2012). Data are collected using the forms for enrolment, and follow-up of families is served by the family health teams and community health agents. It contains data on socio-economic characteristics; health (morbidity); residences of households and their individuals; and medical follow-up data on priority groups such as pregnant women, diabetics, hypertensives, and leprosy patients having tuberculosis, and children younger than 2 years (the mother’s name and address, age of the child, date of death, and cause of death). In addition, medical and nursing consultations, request for additional examinations, referrals, as well as notification of some diseases, for example, pneumonia in children younger than 5 years are recorded.

### SISLAB

Brazil has a national network of public laboratories, the National System of Public Health Laboratories [Sistema Nacional de Laboratórios de Saúde Pública (SISLAB)]. The laboratories are organized hierarchically (national, regional, state, and municipal level) by the degree of complexity of activities, in accordance with the principles of SUS, related to health surveillance including epidemiological surveillance, surveillance in environmental health, sanitary surveillance, and medical assistance (da Saúde, 2004). In 2008, the MoH, aiming to improve laboratory information through the General Coordination of Public Health Laboratories (CGLAB) and DATASUS, elaborated the Laboratory Environment Management System [Gerenciador de Ambiente Laboratorial (GAL)]. GAL is a free software with its own communication patterns, distributed, robust and flexible architecture, and multi-platforms (Jesus et al., 2013; Júnior et al., 2017). The national module of the GAL manages, monitors, and concentrates the results of the laboratory tests informed by the State Modules of the following six areas: Medical Biology, Environmental and Worker Health, Animal, Quality Control, Management, and Quality and Biotechnology. Therefore, GAL is a computerized system applied to the examinations and tests of samples of human, animal, and environmental origins, following the protocols of the MoH.

GAL sends laboratory test results from suspected or confirmed cases of Compulsory Notifications (flu, tuberculosis, leishmaniasis, dengue, zika, yellow fever, pertussis, and meningitis, among others) to the SINAN. It also contains data on viral hepatitis markers, serological diagnosis of HIV, tumor markers, diagnosis of zoonosis and related biological factors, analysis of water quality in health facilities and environmental health surveillance service (Jesus et al., 2013; Júnior et al., 2017). The SISLAB-GAL data contribute decisively to surveillance in Brazil, but its integration with other social-economic information (for example, data from CadUnico) and health care data (for example, SIM/SIH) would allow to conduct several epidemiological studies.

### NOTIVISA

The National Notification System for Health Surveillance [Sistema de Notificações em Vigilância Sanitária (NOTIVISA)], created in 2008, is an online computerized information system of the National Sanitary Surveillance Agency (ANVISA) that receives spontaneous reports of suspected cases of Adverse Drug Events. It covers the Brazilian territory and is considered the largest and most important repository of Adverse Drug Events data from the National Pharmacovigilance System (SINAF) of the country (de Vigilância Sanitária, 2008). NOTIVISA allows the obtaining and circulation of information on health problems to users, sudden or undesirable effect, and/or malfunctions related to health products marketed in Brazil. The NOTIVISA system has enabled adoption of adequate measures of control, safety alerts, besides providing information to update the existing legislation and/or to propose new legislation as well as sanitary recommendations for the adoption of measures that ensure the protection and health promotion of the population (Branco et al., 2015).

Data from NOTIVISA have been used in studies to investigate the occurrence of reports related to health products in the post-marketing phase, such as the occurrence of Adverse Events and Technical Complaints related to the use of a vascular catheter (Oliveira and Rodas, 2017), to describe the adverse events related to healthcare products that resulted in death in Brazil (Maia et al., 2018).

### SNGPC

The National System for Management of Controlled Products [Sistema Nacional de Gerenciamento de Produtos Controlados (SNGPC)], implemented in late 2007 and early 2008, is a sanitary surveillance information system that captures dispensing movement data (inputs and outputs) of the drugs subject to the special control as well as antimicrobials and updates in pharmacies and private drug stores in the country. The SNGPC main objectives include: to monitor the dispensation of drugs and narcotics, and psychotropic substances and their precursors; to optimize the book keeping process; to allow monitoring of prescription habits and consumption of controlled substances in a given region to propose control policies; to collect data that allow the generation of up-to-date and reliable information for the National Health Surveillance Service (SNVS) for decision making; and to streamline the actions of health surveillance (de Vigilância Sanitária, 2010)

The data feeding the system comes from the prescription of qualified medical professionals, retained at the time of dispensing the drug in the pharmaceutical establishment, and invoices for the purchase of medicines suppliers. The main operational actors of the SNGPC are the pharmacists in charge of the pharmacies and drug stores, and SNVS health surveillance professionals. SNGPC has been used in studies to examine the consumption of appetite suppressant drugs (Mota et al., 2014), the consumption of psychotropic anorectic drugs (Martins et al., 2012), and the frequency as well as distribution of the consumption of benzodiazepine anxiolytics in private pharmacies and drug stores (Azevedo et al., 2016).

### PFPB

The Popular Pharmacy Program [Programa Farmácia Popular do Brasil (PFPB)] was created in 2004, within the scope of the SUS, to expand access to medicines for the most common diseases among citizens. One of the objectives of the program was to favor low-income people by making treatment feasible in the face of the high price of medicines. It also supports the population of the private health network as an alternative, since they have access to medicines with prices more affordable. PFPB was also aimed to contribute to the reduction of the expenses generated by the purchase of medicines and minimize the expenses of the SUS with hospitalizations that are caused by the abandonment of the treatment (Inocencio and De Vivo, 2011).

The PFPB developed two axes of action: the own network of public Popular Pharmacies (rede Própria) and accredited private retail pharmacies of PFPB (PFPB-E, “Aqui Tem Farmá-cia Popular” or ATFP). Popular Pharmacies, operating since 2004, have a list of 112 medicines which are dispensed at cost representing a reduction of up to 90% of the market value. The PFPB-E, considered as an expansion of PFPB in partnership with pharmacies and drugstores of the private network, was created with the objective of expanding the coverage of pharmaceutical assistance and promoting the integrity of health care (Coelho Filho et al., 2004). In this modality, the MoH subsidizes 90% of the reference value for diseases, such as dyslipidemia, Parkinson’s, glaucoma, osteoporosis, rhinitis, contraceptives, and geriatric diapers. As of 2011, with the creation of “Health Without Price” (“Saúde Não tem Preço”), the two axes of action started to count on free medicines for asthma, diabetes and hypertension. It has the potential for linkage since it contains tax number (Cadastro de Pessoas Físicas [CPF]) of individual patients (Coelho Filho et al., 2004).

### SINITOX

The National Toxic-Pharmacological Information System [Sistema Nacional de Informações Tóxico-Farmacológicas (SINITOX)] was created in 1980 and is linked to FIOCRUZ. It is responsible for the collection, compilation, analysis, and dissemination of cases of intoxication and poisoning registered by the National Network of Information and Assistance Centers Toxicological—RENACIAT. RENACIAT is currently composed of 36 units located in 19 states and the federal district which provide information and guidance on the diagnosis, prognosis, treatment and prevention of intoxications, as well as on the toxicity of chemical and biological substances and the risks they cause to health (Bortoletto and Bochner, 1999).

The differences in structure, setup, and content of all these different databases can lead to significant challenges in use of these data for HTA and decision-making. In addition, there are considerable challenges regarding the lack of governance. Most often, there are poor or no standards for collaboration; there is a lack of incentives for data sharing; and there are issues with regard to patient consent, privacy, and data security that may severely hamper access to such data. As a result, the costs for data protection would be very high to comply with relevant regulation (Annemans, 2017).

Despite the availability of all these SUS and related databases, there are still key challenges in the use of secondary data for HTA, pharmacovigilance, and supporting decision-making. Data linkage is one of the available approaches that can be used to mitigate lack of integration and standardization observed in such databases. Record linkage can help generate useful and high quality data sets to conduct research, and support formulation and evaluation of public policy. However, linking these databases is not a trivial task mainly due to the lack of common key identifiers amongst all the databases, as well other technical issues related to data quality, standardization, availability, and volume (number of records). **Table 2** summarizes some attributes encountered in most of these databases, which are potential candidates for linkage purposes.

**Table 2:** Potential Linkeage Attributs amongst SUS databases.

Attribute	Meaning	Databases
Name	Full Name	CadUnico, BFP, SIM, SINAN, SINASC, SIH-SUS, SIA-SUS (APAC-SIA), SISMAMA, SIAB, SISLAB-GAL
Mother’s name	Full Name	CadUnico, BFP, SIM, SINAN, SINASC, SIH-SUS, SIA-SUS (APAC-SIA), SISMAMA, SIAB, SISLAB-GAL
Data of birth	Date, Month, Year	CadUnico, BFP, SIM, SINAN, SINASC, SIH-SUS, SIA-SUS (APAC-SIA), SISMAMA, SIAB, SISLAB-GAL
Municipality Code	7 Digit Numeric	CadUnico, BFP, SIM, SINAN, SINASC, SIH-SUS, SIA-SUS (APAC-SIA), SISMAMA, SIAB, SISLAB-GAL
Sex	Male/Female	CadUnico, BFP, SIM, SINAN, SINASC, SIH-SUS, SIA-SUS (APAC-SIA), SISMAMA, SIAB, SISLAB-GAL

## Data Linkage

Data linkage, also called record linkage, is the process of combining records about the same individual or entity from two or more different data sources (Winkler, 2006; Jurczyk et al., 2008a) or the process of identifying duplicate records in the same data set (Jurczyk et al., 2008a). In principle, record linkage problem consists of developing a classifier that categorizes record pairs as “linked” or “non-linked” with reasonable accuracy (Jurczyk et al., 2008a). It enables the aggregation of data not available in a single data set thereby supplementing information on an individual with information from other data sources, validating information collected in one data source, or to de-duplicate records within a single data source (Winkler, 2006; Jurczyk et al., 2008a). Record linkage also has additional applications, such as building longitudinal profile of individuals and case-identification in capture-recapture studies (Sayers et al., 2015).

There are two main types of linkage algorithms: deterministic and probabilistic. Deterministic linkage methods vary from a one-step procedure using a single unique identifier or a set of several attributes (called “exact” deterministic linkage) to step-wise algorithmic linkages involving a series of progressively less restrictive steps to allow variation between record attributes (called “iterative” deterministic linkage). A record pair is classified as “linked” if it meets the criteria or parameters at any step; otherwise is classified as “non-linked” (Dusetzina et al., 2014). Probabilistic linkage methods, on the other hand, takes advantage of differences in the discriminatory power of each attribute and apply calculation of similarity scores, as well as decision rules, to classify record pairs as linked, potentially linked (treated as dubious records in most linkage tools) and non-linked (Newcombe et al., 1959; Fellegi and Sunter, 1969; Dusetzina et al., 2014). It can also deal with some inconsistencies between records with missing data, i.e., it has the capacity to link records with errors in the linking fields (Dusetzina et al., 2014).

**Figure 1:**
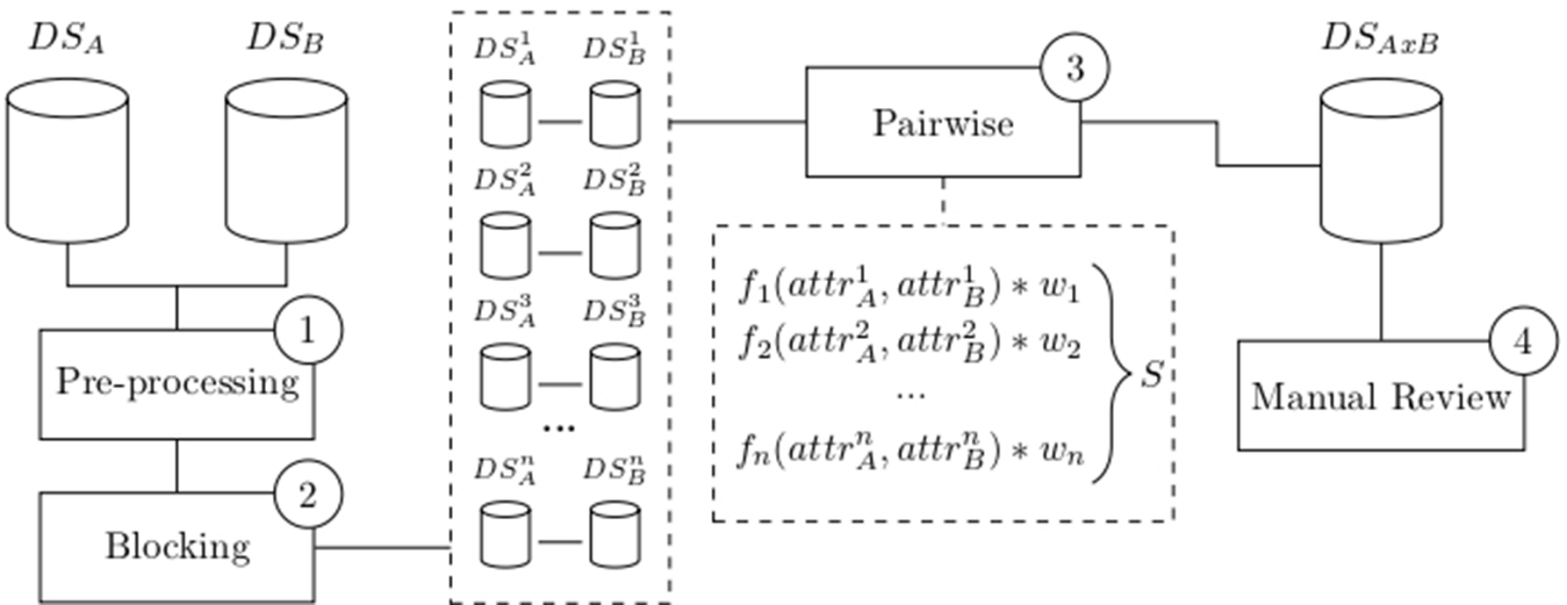
Common flowchart of a data linkage tool: Raw data are pre-processed (1) and split into smaller blocks (2). Pairwise comparison is performed among records within similar blocks using functions that produce a similarity measure for each attribute. A weighted vector is then used to average each individual similarity measure into a single score S (3). Manual review the dataset generated after the pairwise comparison is optionally performed (4). Data source A (DS_A_), data source B (DS_B_), number of blocks (n), attributes (attr), weights (w), score (S), and linked data DS_AxB_.

Since its introduction by Newcombe (Newcombe et al., 1959) and mathematical formalization by Fellegi and Sunter (Fellegi and Sunter, 1969), several variations of record linkage and computerized tools have emerged to meet different requirements and challenges, such as accuracy, speed, and scalability. Many of these tools have a general purpose, allowing a combination of existing configurations and methodologies (Camargo and Coeli, 2000; Elfeky et al., 2002; Christen et al., 2004; Christen, 2008; Schnell et al., 2009; Pita et al., 2018). While most of these methods are probabilistic, some of them apply a combination of deterministic and probabilistic linkages (called “hybrid” methods) (Pita et al., 2018). In general, a successful linkage processing involves several main steps: pre-processing, blocking and indexing, field comparison, weight vector classification, and accuracy assessment (Christen, 2008) as depicted in **Figure 1**.

The pre-processing step involves data cleansing and standardization whereby incomplete and incorrectly formatted data is converted into well-defined, consistent form (Christen et al., 2004; Christen, 2008). Specific approaches to deal with missing data can be applied at this step to i) remove missing fields or entire records or ii) impute missing values based on standard or calculated values. Pre-processing may also involve anonymization using different privacy-preserving techniques, such as Bloom filters (Inan et al., 2008; Pita et al., 2018), to protect sensitive data from disclosure and unauthorized use.

Executing a linkage routine between data sets A and B will result in a number of field comparisons defined by the product |*A*| * |*B*|. In a big data context, these numbers make pairwise comparisons impractical and lead to a number of infrastructure, data processing, and data analysis challenges (Peek et al., 2014; Harron et al., 2017). To circumvent scalability challenges over big data sets, different approaches have been used in the literature, such as parallelism/distribution and blocking (or indexing) strategies, as well as their combinations (Christen, 2008; Pita et al., 2018). Other initiatives have also proposed the use of cluster-based platforms, multi-processors or graphics processing units (GPUs) (Boratto et al., 2018; Pita et al., 2018). Blocking and indexing step generates pairs of candidate records pertaining to the same comparison blocks (Christen, 2012). These methods drastically decrease the number of candidate record pairs to a feasible number thereby speeding up the linkage performance over big data sets while still maintaining linkage accuracy. Several indexing techniques used in linkage solutions are well described in the literature (Christen, 2012).

**Figure 2:**
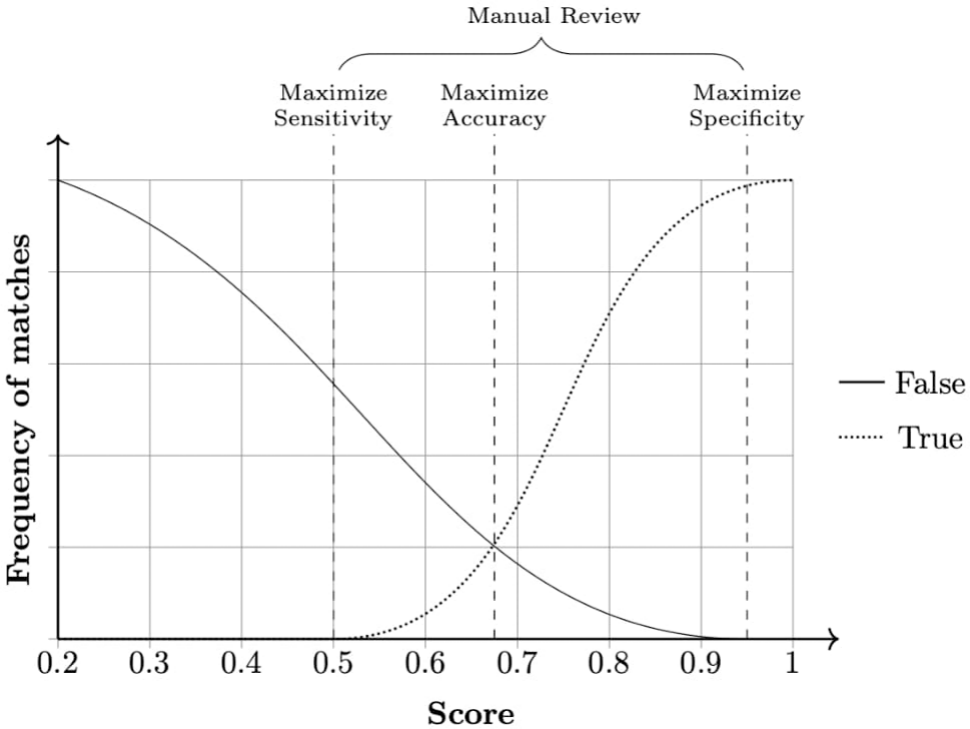
Example of a frequency analysis of a data set produced by the linkage pipeline. Three cut-off points can be chosen according to specific needs. Any pair in between sensitivity and specificity cutoffs is considered a dubious match and thus passed for manual review. When manual review is not possible, a common approach is to choose a cut-off point that averages sensitivity and specificity, maximizing accuracy.

The field comparison step involves using several functions to measure the similarity of attributes for each record pair. The choice of the functions is dependent on the content of the field: string comparison functions are used for names and addresses whereas numerical comparison functions are used for fields, such as date, age, and numerical values (Christen, 2012). Once a vector of numerical similarity values is calculated for each record pair, the candidate record pairs are classified as linked (i.e., candidate pairs that are linked deterministically or probabilistically by the linkage software), non-linked or possibly linked, based on one or more cutoff (threshold) points, in the weight vector classification step as shown in **Figure 2**.

During the final step—accuracy assessment—evaluates the linkage algorithm and the quality of the linkage (i.e., it estimates rates of linkage errors: missed matches and false matches).Linkage accuracy is often assessed using a gold standard dataset where the true match status of each pair of records is known. Comparing the probabilistically linked dataset to the gold-standard dataset will identify true matches, true non-matches, false matches, and missed matches. Hence, measures of linkage quality such as sensitivity, positive predictive value, and F-measure can be easily derived. When gold standard dataset is not available,alternative approaches such as sensitivity analysis, comparison of characteristics of linked and non-linked data, and identification of implausible matches could be used to quantify the rate of linkage errors (Christen, 2008).

## Data Linkage Initiatives in Brazil

In the last decade, the use of big data for research in Brazil has increased substantially due to several factors: data access, creation of research groups and data centers, development of efficient record linkage tools, and international research collaborations, among others. In this section, we describe three data centers specialized in the use of big data as well as development of record linkage tools:

### CIDACS

The Centre for Data and Knowledge Integration for Health [Centro de Integração de Dados e Conhecimentos para Saúde (CIDACS)] is a data linkage center managed by the Oswaldo Cruz Foundation (FIOCRUZ),´ officially launched in December 2016 and located in Salvador, State of Bahia, Brazil. It houses the 100 Million Brazilian Cohort (Pita et al., 2018) and is responsible for housing other large databases, such as SINAN, SIM, and SINASC, as well as the development of other new and innovative studies using these large databases. An agreement signed between the Ministry of Social Development (MoSD), FIOCRUZ, UFBA, and the University of Brasilia (UnB); the MoSD permitted the acquisition, after ethical approvals, of a copy of CadUnico and BF payments from 2004 to 2015. Negotiations with the MoH, in particular, the Department for Health Information (DATASUS) granted CIDACS copies of SINASC, SINAN and SIM from 2000 to 2015. Copies of SIH, SISVAN, and “Minha Casa Minha Vida” (housing program) are also available, whereas access to other databases, such as “Cisterns” (Wells), is in negotiation at the time of writing this paper.

The center operates with a strong governance; an advanced data platform comprising the computational infrastructure needed for receipt, storage, curation, and integration of large databases, and extraction of data sets for specific analysis; and a physical structure carefully designed to give full physical protection for the data when handling non-anonymized data sets as well as to manage access and analysis of de-identified or anonymized data sets. All standard operation procedures (SOPs) for data manipulation, cleaning, linkage, and for meta-data production are being defined according to international standards. In the past few years, CIDACS has developed algorithms for data anonymization and data linkage including the two linkage algorithms (“AtyImo,” a tool used in less safe environments where the identifier information must be masked or anonymized, and “CIDACS-RL,” which is used under extremely safe environments without masking). Linkage using both tools were already validated and, for the optimum threshold (best trade-off of sensitivity and specificity), the accuracy of both algorithms is above 90% (Pita et al., 2018).

### Minas Gerais

The research group at the Federal University of Minas Gerais had also made significant achievement in record linkage. In partnership with the MoH’s team, the team has been working on the National Health Database Centered on Individual: a 15-year cohort of individual-level historical data, preserving patient privacy, integrating SIH, SIA, SIM, SINASC, and SINAN (Guerra et al., 2018). It will allow researchers to generate real-world evidence using clinical, pharmacological, and pharmacoeconomic studies.

The group has also developed a parallel deduplication algorithm, called FER-APARDA, using probabilistic record linkage, as well as PAREIA (Santos et al., 2007; dos Santos Filho, 2008). PAREIA’s crucial contributions are two-fold: 1) the proposed blocking scheme uses predicates from fields or portions of them, making a junction of disjunctions to prevent input errors to separate true matches from the right blocks. 2) The use of high-performance computing techniques and programming languages to guarantee its suitability to big data scenarios. This initiative has enabled several scientific investigations, such as the study of a criminal network by identifying distinct offenders on a graph-based police event database (dos Santos Fraga, 2009).

### Rio de Janero

The research group at the State University of Rio de Janeiro has made substantial contribution on data science and record linkage in Brazil, including the largely used probabilistic record linkage tool based on Fellegi-Sunter model, RecLink (Camargo and Coeli, 2000; Camargo and Coeli, 2006). Reclink has been used in many epidemiological studies by academic institutions and the MoH which were mostly published in *Reports in Public Health*,^1^ a scientific repository maintained by the Oswaldo Cruz Foundation. They have also employed record linkage on administrative databases to study mortality rate on patients submitted to high complex cardiology procedures (Migowski et al., 2011) and to assess the under notification of tuberculosis cases in Brazil (Oliveira et al., 2012b).

Main contributions of this group, beyond the reported linked databases, comprise the use of the Expectation Maximization (EM) algorithm to predict the best settings for model tuning (Junger, 2006), the use of phonetic code in blocking step (Coeli and Camargo, 2002), and the rule-based matching. Recent work on the development of an open source version, the OpenRecLink (de Camargo and Coeli, 2015) has provided a multi-platform solution suitable to international users. Additional efforts have been made to define a cut-off point on probabilistic record linkage results (Verzinhasse Peres et al., 2014) and building a data warehouse for the integration of three Brazilian health information systems concerned with the production of ambulatory and hospital procedures for cancer care, and cancer mortality: SIH-SUS, APAC-ONCO, and SIM (Freire et al., 2015).

## Record Linkage Tools Developed and/or Used in Brazil

In the past decade, the use of secondary data for research in Brazil has grown substantially. This is due to several factors: availability of large data sources, development of efficient linkage tools, legislation in Brazil favoring the used of secondary data for Brazil, the need to evaluate several public policies, and international collaborations, among others.

### RecLink

RecLink (RecLinkIII) is an open source C++ based probabilistic linkage algorithm specifically developed for the Portuguese language phonetics (Camargo and Coeli, 2000). Its flexible Graphical User Interface (GUI) allows the user to customize the tool and read different data sets easily. The interface is also used to define the behavior of the tool, such as the pre-processing steps, blocking and matching parameters (Camargo and Coeli, 2006). RecLink uses a custom format for input, which means the original data sets first have to be converted to standard formats to be linked. It has functions related to the standardization of common fields, including manipulation of names (case sensitive conversions, preposition and accent removal in names, and removal of commas and punctuation marks), standardization of date formats, and correct classification of missing values. Standardization of character attributes, such as date of birth and sex, is performed using the dBASE database manager, whereas a Soundex code developed for this purpose is used for strings, such as names and municipality (Camargo and Coeli, 2000; Camargo and Coeli, 2006).

RecLink performs record linkage in two steps: 1) blocking step, which separates the two data sets to be linked into smaller data sets according to the configuration provided by the user and 2) matching weight calculation and pairwise comparison of records that belong to the same block (Camargo and Coeli, 2000). The blocking stage can be conducted in one-step procedure using a single attribute, such as municipality(Paixao et al., 2018) or multi-step procedure using different combinations of attributes (Capuani et al., 2014). The Levenshtein string comparator is used to compare names; it is defined as the minimum number of insertions, deletions, or substitutions necessary to change one string into another (the values varying between 1, perfect similarity and 0, total disagreement). RecLink uses three different weight systems that can be selected by the user: 1) the pure and simple comparison, which only classifies records as matches if their attributes are strictly identical; 2) the character sequence comparison, which evaluates each pair of attributes from both records and returns how many different characters they have; and 3) the fuzzy comparison, which returns a normalized score consisting of the size of the longest common sequence of characters divided by the size of the longest attribute. The default m-probabilities and u-probabilities of 0.9 and 0.1, respectively, are often used (Oliveira et al., 2012a; Paixao et al., 2018).

RecLink is the most popular linkage tool in Brazil that has been used in several population linkage-based studies (Oliveira et al., 2012a; Capuani et al., 2014; Paixao et al., 2018). It also has several functions for other applications, such as de-duplication, standard query language (SQL) exporting, and frequency tables calculation. The software is available for free use and licensed under GPL (Camargo and Coeli, 2000; Camargo and Coeli, 2006).

### Python Linkage Algorithm

Python linkage algorithm (PLA) is a Python based deterministic algorithm developed for passive data collection with cohorts of HIV-infected patients at FIOCRUZ Rio de Janeiro. The tool aims to maximize accuracy and to minimize the need for clerical (manual) review in data linkage (Pacheco et al., 2008b). It was primarily implemented to assist in retrieval of information on the vital status of people living with HIV/AIDS (PLWHA) who are lost to follow-up in two large urban HIV/AIDS cohorts: Rio de Janeiro cohort database (Schechter et al., 1994) and TB-HIV in Rio (THRio) (Pacheco et al., 2011). The Rio de Janeiro cohort database was originally designed to validate the WHO-HIV staging system in a developing country, whereas the THRio cohort was designed to assess the impact of implementing isoniazid prophylactic therapy among HIV-positive patients with indications for prophylaxis in Rio de Janeiro (Pacheco et al., 2011; Saraceni et al., 2014). PLA has also been adapted to cross-reference PLWHA public databases to both tuberculosis and AIDS cohort databases (Pacheco et al., 2008a; Grinsztejn et al., 2013).

The algorithm has a hierarchical structure and correlates records using exact comparisons. It allows for specific errors in names and dates, measured by means of phonetic codes and a string similarity score based on a recursive longer common substring algorithm, implemented in the “difflib” library from Python, which helps dealing with specific differences between sequences and dates. PLA runs both in a fully automated procedure (PLA-FAP) and in association with clerical/manual review of records that are not classified as true matches or non-matches (PLA-MR). Patient name, mother name, and date of birth are used as matching fields with parameter estimates obtained with the Expectation–Maximization algorithm. Date of birth is allowed to have only one digit mistake in any position or the common swap between day and month (only if they were exactly the same but swapped) (Pacheco et al., 2008b). The Levenshtein distance string comparator measure is used to compare the field name and mother name (Fonseca et al., 2010). The algorithm uses score values chosen empirically during its development using different data sources (Pacheco et al., 2008b).

The combination of these measurements and score values determine several levels of inclusion and exclusion, called automatic codes, which depends on how much information is available or missing. Records with complete information are treated independently from records with missing information. Whenever a pair of records is neither automatically included nor automatically excluded by the criteria, this pair is kept in the final merged database, marked as an unresolved pair for possible further manual review. The algorithm is hierarchical in the sense that lower codes mean more similar records hence perfect matches, but codes used for records with full information, even if higher, are more robust than codes for missing records. The algorithm is not “greedy”: the same record in the test database linked with a lower code (exact match) to one record could also be linked to another one with a higher code (poor match). This feature is useful in dealing with databases with one-to-many relations, for example in the case of tuberculosis surveillance databases (Pacheco et al., 2008b; Fonseca et al., 2010).

PLA has been validated using several cohorts Pacheco et al. (2008b); Pacheco et al. (2011); Saraceni et al. (2014); de Paula et al. (2018) and has comparable accuracy to RecLink, which intrinsically require manual review, and outperformed RecLink significantly in the presence of incomplete data without manual inspection (sensitivity: 98.4% for PLA versus 94.6% RecLink, p *<* 0.05) Pacheco et al. (2008a, 2008b, 2009, 2011); Grinsztejn et al. (2013).

### AtyImo

AtyImo was developed by UFBA and CIDACS between 2013 and 2016 to support a joint Brazil–UK project aimed at developing large population-based cohorts. It was written in Python, freely available on Github, and runs distributed over Spark (Pinto et al., 2017; Pita et al., 2018) or in parallel over CUDA over hybrid (multicore+multi-GPU) architectures. It implements a pipeline comprising data pre-processing (cleansing, standardization, blocking, and anonymization), pairwise comparison and matching decision, and accuracy assessment (Pita et al., 2018).

Data pre-processing in AtyImo is responsible to clean and standardize names, filling null/missing fields with default values, and remove duplicate records. Blocking is based on different predicates built with five linking attributes (name, mother name, date of birth, sex, and municipality). In an effort to reduce errors due to typos or missing values that could lead records being inserted in wrong blocks, AtyImo uses a predicate of attributes in its blocking stage. Anonymization is based on Bloom filters, which guarantee privacy-preserving requisites related to sensitive (identifiable) data, allowing AtyImo to run within less protected environments, if needed. A Bloom filter is a 128-bit vector in which bigrams (pair of characters) are represented as 0 or 1 depending on some hash functions. It is useful to reduce the effort during pairwise comparison; instead of comparing strings directly, one can compare binary vectors using the Sørensen’s Dice similarity function (Pita et al., 2018). Dice is defined as: Dice = (2 h)/(a + b), where h is the total of 1’s at the same positions in both filters, and a and b are the total of 1’s in the first and second filters, respectively. A Dice = 1 means filters are completely equal, decreasing to 0 (zero) depending on existing differences. The current implementation normalizes Dice indices between 0 and 10.000. Dice costs less to compute than other editing distance functions, improving AtyImo’s speed (Pita et al., 2018).

AtyImo implements a two-round linkage step in which a mixture of deterministic and probabilistic methods can be used together to generate high accurate data marts (domain specific data). The weights of each attribute are determined by the amount of bits they occupy in the Bloom, meaning that important attributes, such as name will have a larger bloom length over less important attributes. In the hybrid approach, categorical attributes are matched exactly whereas names and dates (both more prone to errors) are probabilistically classified as: exact (Dice = 10,000), strong (10,000 ≥ Dice ≥ 9,000), weak (9,000 *>* Dice ≥ 8,000), and unpaired (8,000 ≤ Dice). This approach results in some flexibility in the combinations of exact and approximate comparisons. As a result, three output data sets are produced: true positive (TP) pairs, true negative (TN) pairs, and “dubious records” [false positive (FP) and false negative (FN) matches]. This classification is based on upper and lower cutoff points representing boundaries for TP and TN matches, respectively. Further analysis of the cutoff points is performed to retrieve more true (positive and negative) pairs and an iterative second round analysis of dubious pairs, shifting these points in each iteration, is conducted to retrieve additional records into these two groups (Pinto et al., 2017; Pita et al., 2018).

Finally, accuracy assessment can be performed manually based on gold standards (when existent) to certify small data marts or automatically based on supervised machine learning methods (Pita et al., 2017) in big data marts. Supervised methods use the same data produced during the accuracy assessment of previous linkage to fit a model that can be later used to classify new records. AtyImo, in comparison to previous linkage tools freely available, has reasonably better accuracy and shorter execution time with a major advantage to scale upward to huge databases (Pita et al., 2018). The current version of AtyImo based on the NVIDIA’s CUDA library is able to probabilistically link databases of up 80 million records in around 60 s over multiple GPU architectures (Boratto et al., 2018).

### CIDACS-RL

CIDACS-RL, created at CIDACS in Salvador, is a Java-based search engine indexing linkage tool. It was developed to mitigate accuracy and scalability challenges in linking huge administrative electronic health and socioeconomic data sets, in the order of millions of records, stored within the center. To achieve this, instead of using usual blocking strategies, CIDACS-RL uses indexing, query and scoring modules provided by Apache Lucene (Białecki et al., 2012) and inverted index and term frequency-inverse document frequency (TF-IDF) to reduce the number of comparisons. The TF-IDF weight is composed by the normalized TF (the number of times a word appears in a document divided by the total number of words in that document) and the IDF (computed as the logarithm of the total number of documents divided by the number of documents where the specific term appears).

Within the CIDACS environment, all data sets are submitted to data cleansing and quality assurance processes after entering data linkage step. Those processes guarantee that linkage attributes are standardized and cleansed. Similarly to other methods, CIDACS-RL performs record linkage in two steps. If two databases A and B were to be linked and | · | denotes the number of records in a given database, assuming |*A*| *>* |*B*| (i.e., A is the largest database: the indexing module take as input the linkage attributes from data set A (larger data set) and builds an index A_i_ (Białecki et al., 2012). A challenging issue in linking huge data sets is to reduce the number of pairwise comparisons, therefore, CIDACS-RL uses the query module as a blocking stage. Hence, instead of comparing each record of data set B with every record of data set A, CIDACS-RL query a small subset of similar records from A_i_ and apply comparison functions on them.

As Apache Lucene provides different query types, CIDACS-RL uses a mixture of queries functions (exact, semi-exact or fuzzy) to overcome different errors expected to exist in data linkage attributes (Białecki et al., 2012). Exact query takes each linkage attribute as a parameter and returns only records in which every attribute is equal to those used for querying. Semi-exact query is a modification of exact query composed of an arrangement of n1 linkage attributes, hence, enabling retrieval of candidate pairs where only one attribute is different between the query record and result pairs. Unlike exact and semi-exact queries, fuzzy query allows differences on any number of attributes. Each query function (exact, semi-exact or fuzzy) takes each record in data set B to query in A_i_ and returns a set of similar records based on TF-IDF.

Since some attributes may have semantic meaning which the TF-IDF does not account for, CIDACS-RL relies on a custom scoring function tailored for Brazilian data sources to compare record pairs. This function is based on different metrics and approaches, depending on the type of attribute. CIDACS-RL supports four kinds of attributes: string, categorical, date, and IBGE municipality code. The IBGE code is a seven-digit numeric code where the first two digits represent one of Brazil’s 27 states, the following four digits represent one of 5,570 municipalities and the last digit is used for verification purposes. Next, each record in data set B which was used as source for the query is compared with all records retrieved from A_i_ and returns the most similar record based on the custom scoring function. The function returns all pairs matched along with the score obtained; if any record with a score greater than the threshold is found on exact or semi-exact queries, the pair is added to the resulting set and fuzzy query is not executed.

### FRIL

Fine-grained Records Integration and linkage Tool (FRIL) is a Java based tool providing a set of highly customizable functions. Data integration (or reconciliation) is supported through different merging and splitting functions. It uses searching methods to determine which pairs of records will be compared from both data sets to be linked. FRIL has two different search methods: nested loop join and sorted neighborhood. Nested loop method performs “all to all” comparison, which is the same as no blocking. Sorted neighborhood defines a window limit in a way that records outside the window are not compared, reducing the number of comparisons (Jurczyk et al., 2008a; Jurczyk et al., 2008b).

To compare record pairs, FRIL implements four types of distance functions: edit distance, soundex, Q-gram and equality. All distances are normalized between 0 (total disagreement) and 1 (total agreement). Edit distance consists of the number of changes needed to make both attributes equal. Soundex transforms the attributes to a new form that takes the word sound into account, which can be useful when dealing with attributes, such as names and addresses, which are informed using speech. Equality just assigns 1 if the attribute is equal and 0 otherwise (Jurczyk et al., 2008a; Jurczyk et al., 2008b). Regarding the decision process, FRIL allows for matching weights to be assigned to each attribute by the user. The matching weights are then used to compute a normalized weighted average that is the similarity score for the pair. Similarity score is used to make a decision classifying the pair as match, non-match, or uncertain, according to thresholds defined by the user (Jurczyk et al., 2008a). Uncertain pairs can be later manually labeled as matches or non-matches, if needed. FRIL has been used in Brazilian databases and comparative studies of linkage algorithms, however, the performance in huge data sets was sub-optimal compared others, such as AtyImo (Pita et al., 2018).

### Febrl

Freely extensible biomedical record linkage (Febrl) is a Python-based data linkage pipeline, implementing data cleansing, de-duplication, and pairwise comparison. Its modular architecture contains a handful of functions that can be used in the linkage process. The tool has a graphical interface (GUI) that can be used to customize settings according to the data sets and desired results. Febrl is organized in two main parts: pre-processing and the linkage itself. There is also a data exploration functionality that can be used to visualize the data and make sure it was loaded correctly (Christen et al., 2004; Christen, 2008).

Febrl supports several text-based data set formats: columnar text files, CSV, and SQL databases. Pre-processing comprises data cleaning and standardization through different standard functions for names, dates, and addresses. There is also an implementation of Hidden Markov Models (HMM) for name and address segmentation. For example, a single attribute name can be split into first name and second name. The HMMs can also be applied to addresses to extract multiple attributes (such as ZIP code, house number, city name, and state) from a single one. Febrl also has functions for date standardization, which are useful when two data sets have different date formats (Christen et al., 2004; Christen, 2008).

Pairwise comparison includes blocking methods and multiple functions to compare attributes. Febrl contains seven blocking implementations. They aim to reduce the amount of comparisons, and thus reducing the computational cost of the linkage. Febrl allows for each attribute to be compared using a different comparison function. Hence, it is possible to explore different strategies to find the one that better fits the set of attributes in the given data set; the tool has more than 20 different comparison functions. When comparing two records, each comparison function returns a score for attribute pair. On top of the score produced by each comparison function, it is necessary to use a definitive classifier, for weight vector classification, that generates a single score for the pair. The classification process can be supervised or unsupervised. After the classification step, it is also possible to customize the output of the system. This option allows the user to specify if the output will be one-to-one or one-to-many comparison (Christen, 2008). Similarly, comparative studies on data linkage tools in huge Brazilian databases showed slightly sub-optimal performance compared to RecLink and AtyImo (Pita et al., 2018). **Table 3** compares the five recording linkage tools using different attributes.

**Table 3:** Comparative analysis of existing linkage tools.

Feature	RecLink	PLA	AtyImo	CIDACS-RL	FRIL	Febrl
Deterministic	Pure Comparison	Exact Comparison	Hybrid approach	Exact query	Equality function	Exact comparison functions
Probabilistic	Character Sequence and fuzzy	Automatic codes	Fully probabilistic	Semi-exact and fuzzy queries	Edit distance, soundex and Q-gram	Approximate comparison functions
Blocking	One step (single attribute) and multi-step predicates)	No	Predicates	TF-IDF indexing	Nested loop join and Sorted neighbourhood	Block, Ssorted and fuzzy (bigram)
Anonymization	No	No	Bloom Fliter	No	No	No
Manual review of Dubious records	No	PLA-MR	Second round with adjusted cut-offs	Yes	Yes	Yes
Automated review of dubious records	No	PLA-FAP	Machine learning-based	No	Yes (expectation maximization)	Expected
Open source, freely available	Yes(GPL)	No	Yes	No	Yes	Yes

## Challenges and Opportunities

Linked administrative data sets hold the potential to change the research landscape in the HTA arena, since linkage constitutes a valuable tool for combining individual-level information (biological, behavioral, socio-economical, clinical, and environmental) from different sources. This combined information can be used for population-based research applications with implications for public health, as well supporting public policy decision making.

Administrative data differ substantially from data generated by the academic community. Government data is collected in a logical format over time and refers to the totality of populations or specific groups; academic data is limited in scope, generally collected over a defined period for specific purposes. The use of linked administrative data for research, compared to those of primary data, often has several challenges and limitations. However, it can have several advantages: 1) large sample size, enabling statistical power for stratified analysis, making possible to explore epidemiological questions in different sub-populations; 2) it can help rebuild the prospective characteristic of the data, allowing for longitudinal studies at relatively lower cost with retrospective data; 3) it can also help answer questions that require detailed data on hard-to-reach populations, such as children; and 4) it can also help generate evidence with a high level of external validity and applicability for policy making as it captures the real-world setting. In the area of HTA, administrative data holds the potential to contribute to the development of high-quality and powerful research that furnishes scientific evidence for use by policy makers.

Brazil has made substantial effort to improve the quality of the data collected as part of service delivery. Although huge variation exists between regions and states, most data sets contain incomplete, inconsistent, inaccurate data that vary in content, format, and structure. This has substantial impact on data pre-processing requirements, quality of linked data, and internal validity of research findings from the data (Harron et al., 2017). Although considerable proportion of missing values in non-mandatory variables in a specific data set can be expected, for example education and occupation, key variables are more likely to be complete. In addition, similar variables are recorded in different data sets: a missing variable in one data set can be recorded in another data set; hence, in some circumstances, the linkage process can recover this missing variable.

Importantly, due to lack of unique key identifiers, all linkage tools used in Brazilian data including RecLink, AtyImo, and CIDACS-RL, rely on names, sex, date of birth, and municipality as linkage attributes (Camargo and Coeli, 2000; Pita et al., 2018). Brazilian names are often recorded in different ways, for example, an individual with five names might have only the first and last name recorded in one data set but all the five names recorded in the other data set (Harron et al., 2017; Paixao et al., 2018). In addition, there are misspellings, abbreviations, and punctuation marks with names and municipalities, and different date formats with date of birth which require a time-consuming data cleansing and sometimes sophisticated techniques for standardization. The level of data cleansing performed should take into account preserving the discriminative power of individual identifiers and the ability to distinguish one record from another (Harron et al., 2017). To minimize errors and improve comparison of record pairs, RecLink (Camargo and Coeli, 2000; Camargo and Coeli, 2006) and CIDAS-RL use string comparators and phonetic coding adapted to Brazilian names, whereas AtyImo uses a predicate of attributes in its blocking stage and Bloom filters (Pita et al., 2018).

The availability of huge volumes of data also provides opportunities to explore the effect of interventions or policies on frequent as well as rare health outcomes and in sub-populations including vulnerable groups (e.g., children, woman, ethnic minorities). At the same time, storage, processing, and analysis of such huge data has proved challenging for research institutions despite recent technological advances. The 100 Million Brazilian Cohort, an electronic database comprising individual records of approximately 114 million people (57% of the Brazilian population) is one example. The extensive coverage in the social registry: CadUnico, the availability of individually identified data, and the possibility to link to other health care data sets made it possible to design individual level longitudinal studies to evaluate the impact of BFP and other social protection programs on health and health-related outcomes.

CIDACS, the data center located in Salvador, needed to utilize sophisticated infrastructures for storage and processing of data, yet it took several days to link some data sets. Again, the lack of unique key identifiers in the databases to be linked required the use of techniques (for example, the use of predicates and Bloom filters in AtyImo or search engine indexing and scoring in CIDACS-RL) to reduce computation time at both blocking and comparison steps of the linkage process. Freely available linkage software, such as FRIL, Febril, or RecLink, crashed when attempting to run the linkage to build the 100 Million Brazilian Cohort, even on high-speed computers, prompting the need for developing efficient linkage tools: AtyImo and CIDACS-RL. Furthermore, the large size of the linked data may pose analytic challenges for standard statistical packages, such as SPSS and R, to run advanced statistical methods, for example propensity score matching.

Providing access to and usage of administrative data sets containing personal identifiable information for linkage purposes also presents a range of privacy challenges, mainly with regard to ethical and legal issues in the effort to protect personal data. Processing, linkage, and analysis of data should be conducted in accordance with the principles and criteria designed to ensure individuals’ privacy, data security, and the ethical use of data containing personal information. This could vary from country to country or from center to center. For example, CIDACS’s big data platform utilizes: 1) a combination of physical and virtual environments including the separation of data curation, linkage and analysis, 2) a hierarchical data access policy ensuring that only a specified number of individuals possess the highest level of access to all data elements for treatment purposes, and 3) privacy-preserving linkage tool, for example, AtyImo uses hash functions to anonymize relevant fields before the record linkage stage (Pinto et al., 2017; Pita et al., 2018). The hash functions, in addition to anonymization, help to speed up the linkage process (Pita et al., 2018) despite complicating the process of assessing the similarity between identifiers on different records (Harron et al., 2017). On the other hand, CIDACS-RL uses safe heaven to protect privacy while processing and linking data sets.

In the center’s access and analysis environment, researchers have exclusive permission to access anonymized linked data sets *via* the coded data variables relevant to their field of study, after obtaining approval from the institutional review board. Access to data sets can be in person at the data center or through a Virtual Private Network (VPN), in accordance with sound information security practices. These includes: 1) submission of detailed research project accompanied by favorable ethical opinion and filled forms for the data plan provided by CIDACS to support the linkage and extraction of variables contained in the available databases, which should be restricted to those necessary to answer the questions in the proposed study. Detailed descriptions of the processes to be applied and the analyses to be conducted on the data are also desirable to avoid methodological biases. 2) Signature of the “Terms of Responsibility” related to the access and use of data.

Administrative data are generally collected by government departments or agencies for specific purposes, which contain personal information that may be confidential and/or sensitive, such as data collected for the execution of social programs or health service delivery. The use of these data for secondary use in restricted research requires a case-by-case analysis taking into account the balance between risks and benefits to individuals and the predominant public interest. Linking of records between different data sources, administrative or non-governmental, requires individualized data for the application of record linkage techniques, in the absence of unique identifier in the different data sources. As a result, access, processing, and analysis of data containing personal information for the purpose of research and the generation of evidence for decision making in public health policy require legal basis, physical and virtual security arrangements, exclusive use for a purpose previously specified, appropriate credentials for access, and favorable ethical opinion of the proposed study (Harron et al., 2017).

In Brazil, the Law on Access to Information (LAI) provides guidelines for the organs and entities of the federal public administration to adjust their information management policies by promoting the necessary adjustments to the registration, processing and archiving of documents and information. However, LAI does not address the use of information collected or stored by government for use in research because it is more focused on public transparency. A general law for the protection and processing of personal data including data for research purposes, the Personal Data Protection Bill, processed by the Chamber of Deputies since 2012 was recently sanctioned presidential. The General law on the Protection of Personal Data (Law 13709/2018) determines the rights of citizens to their personal data and the criteria that public and private agents will have to obey in dealing with them. It requires the regulator to request privacy risk reports to make sure that personal data are being safely processed, stored, and accessed. Hence, the law might present challenges for governance and management of the entire life cycle of the data requiring investments in computer infrastructure and specialized personnel, and adherence to good information security practices, to maintain the privacy and confidentiality of personal data. The law, which will come into force in February 2020, is the first Brazilian law on the subject and will establish specific norms for the treatment of personal information for public health research.

## Conclusion

Brazil has high quality of health care records, growing number of linkage centers, and an open, modern attitude toward use of data for research and policy including HTA, supported by a rigorous but enabling legal framework. Despite the technical, infrastructural, and legal challenges with the use of huge secondary data for research, data linkage creates a unique opportunity to conduct large-scale observational studies to generate evidence on the impact of health technologies and health/social policies.

## Author Contributions

LS, MSA, and MLB contributed to the conception and design of the study. MSA wrote the first draft of the manuscript with substantial contributions by other authors on the different sections of the manuscript. All authors have read and revised the manuscript critically for important intellectual content, and approved the final version of the manuscript.

## Funding

This work is part of the The 100 Million Brazilian Cohort project funded by the Wellcome Trust, Grant code: 202912/B/16/Z.

## Conflict of Interest Statement

LS holds research grants from GSK, Wellcome, MRC, NIHR, BHF, and Diabetes UK and is a Trustee of the British Heart Foundation.

The remaining authors declare that the research was conducted in the absence of any commercial or financial relationships that could be construed as a potential conflict of interest.

The handling editor is currently co-organizing a Research Topic with one of the authors LL, and confirms the absence of any other collaboration.

## Footnotes

1
https://scielosp.org/grid/csp/

## Abbreviations

ACR: American College of Radiology
ACS: Agentes comunitários de saúde (”The community health agents”)
Anvisa: Agência Nacional de Vigilância Sanitária (”National Agency for Health Surveillance”)
APAC-ONCO: Autorizações de Procedimentos de Alta Complexidade Oncológica (”The High Complexity Oncology Procedures Authorizations for Oncology”)
APGAR: Appearance, Pulse, Grimace, Activity, and Respiration
ATFP: Aqui Tem Farmácia Popular (accredited private retail pharmacies)
AUC: Area under (receiver operating characteristic) curve
BFP: Bolsa Familia Program (”the Conditional Cash Transfer Program”)
BPAI: Boletim de Produção Ambulatorial Individualizado (”Individual Outpatient Production Periodicals”)
BPA: Boletim de Produção Ambulatorial (”The Ambulatory Production bulletin”)
BI-RADS: Breast Imaging Reporting and Data System
CadUnico: Cadastro Unico
CGLAB: Coordenação Geral de Laboratórios de Saúde Pública (”The General Coordination of Public Health Laboratories”)
CIs: Confidence intervals
CIDACS: Centro de Integração de Dados e Conhecimentos para Saúde (”the Centre for Data and Knowledge Integration for Health”)
CIDACS-RL: CIDACS record linkage
CNS: Cartão Nacional de Saúde (”National Health Card”)
CONITEC-SUS: Comissão Nacional de Incorporação de Tecnologias no Sistema Único de Saúde (“The National Committee for Health Technology Incorporation into SUS”)
COSAC/SACS: Coordenação de Saúde da Communidade/Secretaria de Assistência à Saúde (“co-ordination of community health/health care Secretariat”)
CPF: Cadastro de Pessoas Físicas (“Individual Taxpayer Registry number”)
DATASUS: Departamento de Informática do Sistema, Único de Saúde (“Department for Health Information”)
DO: a declaração de, óbito (“ The Declaration of Death”)
DGITS: Departamento de Gestão e Incorporação de Tecnologias em Saúde (“The Department of Management and Incorporation of Health Technologies”)
DNV: Declaração de Nascido Vivo (“the declaration of live birth”)
DSA: Data set A
DSB: Data set B
DSAXB: Data set A and B linked
DST: Departamento de Ciência e Tecnologia – Decit (“The Department of Science and Technology “)
EM: Expectation Maximization
Febrl: Freely extensible biomedical record linkage
FII: Ficha Individual de Investigação (“Individual Investigation Form “)
FIN: Ficha Individual de Notificação (“Individual Notification Form”)
FIOCRUZ: Fundação Oswaldo Cruz (“The Oswaldo Cruz Foundation”)
FRIL: Fine-grained Records Integration and linkage Tool
GAL: Gerenciador de Ambiente Laboratorial (“the Laboratory Environment Management System”
GPUs: Graphics Processing Units
GUI: Graphical User Interface
HMMs: Hidden Markov Models; IBGE, Instituto Brasileiro de Geografia e Estatística (“Brazilian Institute of Geography and Statistics”) Instituto Nacional de Câncer
ICD: International Classification of Diseases
LAI: The Law on Access to Information
MCMV: Minha Casa Minha Vida (the Housing Program)
MoH: Ministry of Health; NOTIVISA, Sistema de Notificações em Vigilância Sanitária (“The National Notification System for Health Surveillance”)
PAHO/WHO: Pan American Health Organization/World Health Organization (PAHO/WHO)
PFPB: Programa Farmácia Popular do Brasil (“The Brazilian Popular Pharmacy Program”)
PLA: Python linkage algorithm
PLA-FAP: Python linkage algorithm—fully automated procedure
PLA-MR: Python linkage algorithm—manual review
PLWHA: People living with HIV/AIDS
PNI: do Programa Nacional de Imunização (“The National Immunization Program”)
PPV: Positive predictive value
RAM: Random-access memory
RCBP: Registros de Câncer de Base Populacional (“The Population-Based Cancer Registry”)
REBRATS: Rede Brasileira de Avaliação de Tecnologias em Saúde (“the Brazilian Network for HTA”)
RENACIAT: Rede Nacional de Centros de Informação e Atenção Toxicológical (“the National Network of Information Centers and Toxicological Attention”)
RHC: de Registro Hospitalar de Câncer (“The Hospital Cancer Registry”)
ROC: Receiver operating characteristic
SIAB: Sistema de Informação da Atenção Básica (“The Basic Health Care Information System”)
SI-AIU: Sistema de Informação de Apuração dos Immunobiológicos Utilizados (“the Immunization Program Evaluation System”)
SI-API: Sistema de Informação de Avaliação do Programa de Immunização (“the Immunization Program Evaluation Information System”)
SI-CRIE: Sistema de Informação do Centro de Referência para Immunobiológicos Especiais (“the Information System of the Reference Center Ffor Special Immunobiologicals”)
SI-EAPV: Sistema de Informação de Eventos Adversos Pós Vacinais (“the Information System of adverse events following vaccinations”)
SI-EDI: Sistema de Informação de Estoque e Distribuição de Immunobiológicos (“the Immunobiological Inventory and Distribution Information System”)
